# Does in-bed cycling delivered within 48 hours of mechanical ventilation, reduce the occurrence of delirium in critically ill patients: A mixed-methods feasibility randomised controlled trial protocol

**DOI:** 10.1177/17511437251400612

**Published:** 2025-12-08

**Authors:** Jacqueline Bennion, Mark Hudson, Mary Hickson, Victoria Allgar, Bridie Kent, David McWilliams, Daniel Martin

**Affiliations:** 1 NIHR Doctoral Fellow, Peninsula Medical School, University of Plymouth, Devon, UK; 2 Public and Patient Involvement and Engagement Representative; 3 Professor of Dietetics, School of Health Professions (Faculty of Health), University of Plymouth, Devon, UK; 4 Professor of Medical Statistics, Head of Medical Statistics Group and Peninsula Clinical Trial Unit (PenCTU), Peninsula Medical School, University of Plymouth, UK; 5 Professor of Leadership in Nursing, School of Nursing and Midwifery (Faculty of Health), University of Plymouth, Devon, UK; 6 Professor in Critical Care and Rehabilitation, Centre for Care Excellence, Coventry University, UK; 7 Professor of Perioperative and Intensive Care Medicine, Peninsula Medical School, University of Plymouth, UK; 8 Intensive Care Unit, University Hospitals Plymouth, Devon, UK

**Keywords:** critical illness, respiration, early ambulation, delirium, patients

## Abstract

**Background:**

Delirium is a severe neuropsychiatric clinical state presenting as an acute onset of cognitive deficits. Patients receiving invasive mechanical ventilation (IMV), have the highest incidence (50%–80%) of delirium amongst patients admitted to intensive care units. Preliminary data indicates that early mobilisation is associated with reduced delirium in critically ill patients. However, definitive evidence is lacking. Current practice varies due to many barriers to patients, who require IMV, receiving early mobilisation interventions. In-bed cycling may address some of these barriers. This research aims to evaluate the feasibility and acceptability of early in-bed cycling to reduce delirium in critically ill patients.

**Methods:**

This multi-site feasibility randomised controlled trial will evaluate early (⩽48 h following IMV), in-bed cycling as a method of early mobilisation, to reduce delirium. Eighty-four participants will be randomised across three sites in a 1:1 ratio, to receive either early in-bed cycling in addition to usual care or usual care alone. The primary outcome is feasibility (recruitment, retention, intervention fidelity). Secondary outcomes include different methods of measuring delirium, physical function, length of stay, ventilator free days, sedation free days, Richmond Agitation Sedation Scale, adverse events and mortality. Descriptive statistical analyses will be conducted. Hypothesis testing will be used for exploratory analysis of the mechanistic sub-study outcomes. An embedded qualitative interview study will evaluate the acceptability of this research.

**Conclusion:**

This trial has been prospectively registered (ISRCTN74277350) and received full ethical approval (REC reference: 24/SC/0096). The trial opened to recruitment in July 2024. Recruitment will take place across 18-months.

## Background

Delirium is a prevalent and serious neuropsychiatric condition in critical care settings, associated with significant morbidity and mortality.^1^ It is defined as a severe neuropsychiatric clinical state presenting as an acute onset of cognitive deficits for example, inattention, fluctuant levels of consciousness from near-coma to severe agitation and psychotic episodes.^2–4^ Among intensive care unit (ICU) patients, those receiving invasive mechanical ventilation (IMV) exhibit the highest incidence of delirium (50%–80%).^2,5^ From 2022 to 2023, there were 189,141 ICU admissions in the UK, and of these 80,902 patients (43%) received IMV within 24-h of admission.^6^ Delirium is associated with long-term cognitive impairment, poor memory, hallucinations, attention difficulties and poor quality of life (QoL) following discharge from the ICU.^7^ Moreover, a systematic review estimated the substantial economic costs associated with delirium.^8^ Findings suggested that costs were increased by 52% when the development of long-term cognitive impairment as a consequence of delirium was considered. Identifying the causal mechanisms of delirium are challenging due to the number of precipitating (sedation, immobility) and predisposing (age, co-morbidities) factors for the development of delirium.^2,9^ The cerebral metabolic insufficiency hypothesis and severe systemic inflammation have been proposed as plausible theories.^2^ These describe biological mechanisms leading to acute brain dysfunction and thus, the development of delirium. Best practice guidelines suggest a bundle of care involving pharmacological and non-pharmacological interventions (including early mobilisation interventions) to prevent and manage delirium.^10^ There is no standardised definition of early mobilisation. However, mobilisation is described as ‘a type of intervention within rehabilitation that facilitates the movement of patients and expends energy with a goal of improving patient outcomes’.^11^ A recent systematic review and meta-analysis investigated if early mobilisation as a stand-alone intervention or as part of a bundle, prevented or reduced delirium in critically ill patients.^12^ Results showed that early mobilisation as part of a care bundle reduced the risk of delirium by 47% and reduced delirium duration by 1.8 days. There was significant variation of results between trials investigating out-of-bed mobilisation interventions and no significant results for early mobilisation as a stand-alone intervention. However, there was a high level of uncertainty and heterogeneity of interventions and populations. Only six included studies investigated early mobilisation as a stand-alone intervention. Of these, two (one randomised controlled trial, one feasibility trial) included delirium as a primary outcome and neither were sufficiently powered. Many barriers exist to implementing early mobilisation interventions for patients requiring IMV.^13^ In-bed cycling may address barriers such as IMV to mobilising critically ill patients.^14^ However, further evidence is needed to investigate the effectiveness of the intervention as a stand-alone method of early mobilisation.^14,16^ Public advisory group (PAG) members, with experience of delirium and early mobilisation in the ICU (previous patients and relatives), reported that in-bed cycling may improve implementation of early mobilisation for patients receiving IMV, because it can be delivered in bed. Moreover, the public representatives highlighted the value of understanding the experience of in-bed cycling from the perspective of patients, relatives and/or carers.

## Aim

The aim of the FRECycl-D trial is to evaluate the feasibility and acceptability of early (⩽48 h following IMV), in-bed cycling as a method of mobilisation, to reduce delirium in the ICU.

### Trial design

A multi-site feasibility randomised controlled trial (RCT) with an embedded qualitative interview study and mechanistic sub-study. The qualitative interview study and mechanistic sub-study will be detailed in their respective protocols.

## Methods

The SPIRIT checklist (2013) has informed the development of this protocol.^17^ A previous systematic review investigating in-bed cycling as a method of early mobilisation in the ICU guided the development of the in-bed cycling protocol.^18^ The relevant core outcome sets (COS) have guided the selected delirium outcomes and functional outcome measures.^19,20^ The embedded qualitative study will be reported separately. It will inform the trial evaluation including the acceptability of this research from the perspectives of the key stakeholders.

### Setting

Adult patients receiving IMV on ICUs will be recruited into the FRECycl-D trial across three sites:

- University Hospitals Plymouth NHS Trust, Derriford.
- Torbay and South Devon NHS Foundation Trust, Torbay.
- Blackpool Teaching Hospitals NHS Foundation Trust.

### Participants

Eighty-four adult patients who meet the eligibility criteria will be enrolled into the trial within 48-h of IMV being initiated. Eligibility criteria were decided upon in consultation with expert recommendations, the research team and reference to similar trials to optimise comparability of findings. Electively admitted patients were not included due to their known predicted shorter ICU stay. Following review of the site Intensive Care National Audit and Research Centre quarterly reports, discussion with the site teams, trial steering committee and regional network recommendations, the inclusion criterion, expected to remain on IMV, was revised (Table s1, Supplemental File).

#### Inclusion criteria

- Adults (aged ⩾ 18 years)
- Unplanned ICU admissions
- IMV initiated ⩽ 48 h after ICU admission
- Expected to remain on IMV > 24 h (amended from >72 h)

#### Exclusion criteria

- Contraindications to mobilisation
- Known or suspected cognitive impairment and/or learning difficulties
- Plan is for palliation/withdrawal of treatment
- Immobile prior to ICU admission
- Body weight over the device safety limit (⩾135 kg)
- BMI < 18.5 kg/m^2^
- Planned ICU admission
- Pregnancy
- Prisoners

### Randomisation

Following written informed consent or consultee agreement, eligible participants will be randomised using permuted block-randomisation, stratified to site, in a 1:1 ratio, to either the intervention or comparator group. The randomisation module in REDCap provided by the Peninsula Clinical Trials Unit (PenCTU) will be used.

#### Intervention

**Figure 1.**
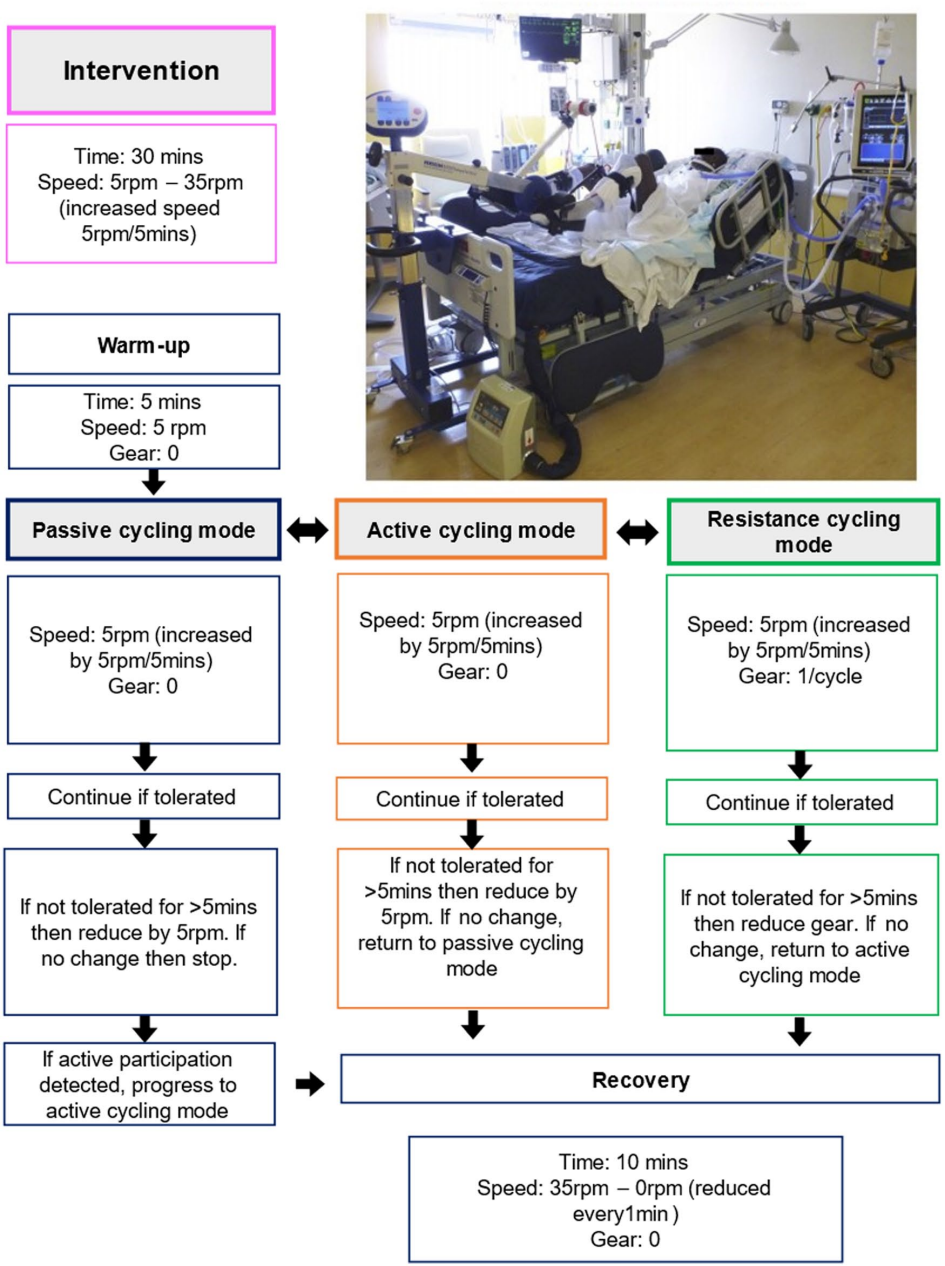
In-bed cycling protocol.

Participants in the intervention group will receive in-bed cycling 5 days per week for a maximum of 14-days from randomisation or until out-of-bed mobilisation commences (whichever comes first). The delivery (days) of the intervention was decided upon in consultation with members of the research team with consideration of available resources and NIHR award collaborators who represent diverse professions and experience in critical care (see Figure 1 below and Figure s1 for details). The intervention will be in addition to usual care. The chief investigator/site physiotherapist/ICU rehabilitation team member at each site will deliver the intervention using an in-bed cycling device (MOTOmed, CE marked with class II medical device status and used as per intended purpose). A safety assessment will be conducted each day prior to commencing the in-bed cycling protocol (Tables s2–s6). The safety assessment has been informed by an international consensus of recommendations for mobilising patients receiving IMV in the ICU.^21^ The recommendations consider in-bed and out-of-bed safety criteria across four categories: neurological, respiratory, cardiovascular and other criteria. These were reviewed by, and agreed upon, with two local expert advisors (DM, RB). The in-bed cycling protocol and intervention safety criteria are described in detail in the Supplemental Material.

#### Comparator

Usual care only. Early mobilisation initiated within 4 days of ICU admission is unusual in UK ICUs. Generally, non-cycling exercise is initiated in patients receiving IMV for example, assisted-limb movement. Currently, the participating sites do not use in-bed cycling within 48-h of IMV.

All participating sites will follow and record a targeted sedation protocol for the intervention and comparator group using the Richmond Agitation Sedation Score (RASS −4 to +2) to minimise confounding factors.

### Outcomes

#### Primary outcomes

The following measures will be collected to determine trial feasibility:

- Recruitment rate (% of participants enrolled vs participants eligible),
- Retention rate (% of enrolled participants who completed the intervention protocol in full excluding deaths),
- Intervention fidelity (% intervention sessions completed in full).
- The acceptability of the intervention will be evaluated by the key stakeholders (assessed by qualitative interviews). The protocol for this will be published separately.

#### Secondary outcomes

A variety of delirium outcome measures have been selected following guidance from the DelCORS study to explore how delirium can be recorded.^19^ Evaluation of these complimentary outcomes will provide important understanding of the methods for characterising delirium for example, duration, severity specifically for patients requiring IMV. These data will contribute towards the selection of the primary outcome measure for a future definitive trial. The timepoints of the listed outcomes are detailed below and Table s7. Please see the separately published statistical analysis plan for details of delirium assessment and analysis of outcomes.

- Occurrence of delirium (CAM-ICU, day 0–14, day-30)
- Delirium free days (CAM-ICU, day 0–14, day-30)
- Duration (days) of delirium (CAM-ICU, day 0–14, day-30)
- Severity of delirium (CAM-ICU-7, day 0–14)
- Physical function (Functional Status Score for the ICU (FSS-ICU), day 14/out-of-bed mobilisation)
- Time to delirium resolution (number of days)
- ICU and hospital length of stay (number of days)
- Ventilator free days (number of days, day 0–30)
- Sedation free days (number of days, day 0–30)
- Daily Richmond Agitation Sedation Scale (RASS)
- Adverse events
- Deaths

### Follow-up

All trial participants will be followed up at 90-days from randomisation. Participants will complete questionnaires measuring quality of life (QoL) and pain (EQ-5D-5L, SF-36), a cognitive screening assessment (MOCA) and assessment of their physical function (6MWT). Their relatives and/or carers will complete a proxy QoL questionnaire (proxy-EQ-5D-5L) and an assessment for the presence of delirium (FAM-CAM).

### Participant timeline

**Figure 2.**
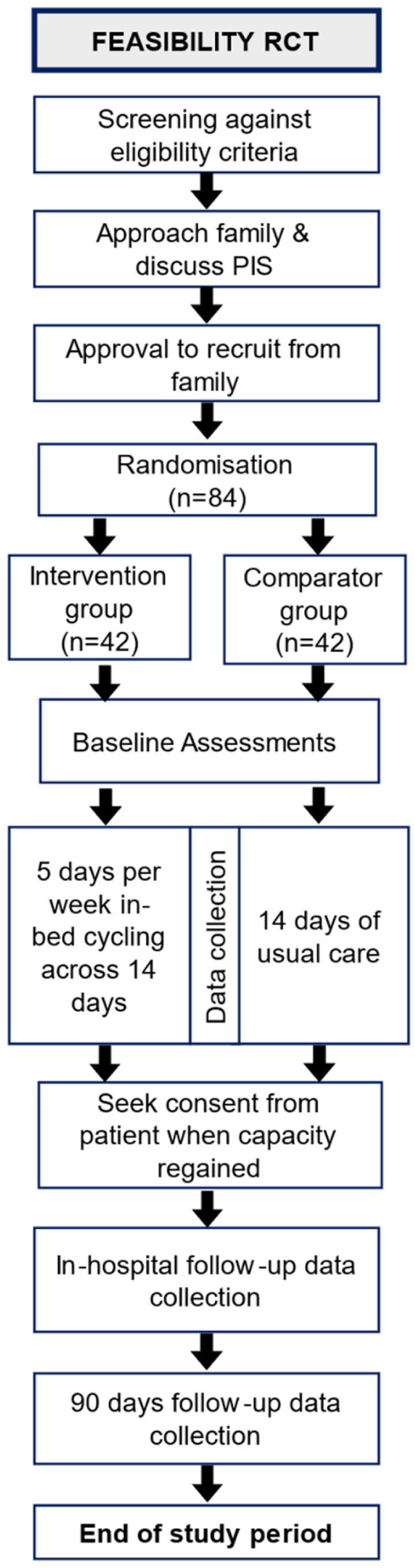
Participant flow.

See Figure 2 below for participant flow through the trial.

### Blinding

Blinding of participants, research and clinical personnel will not be possible due to the visible nature of the intervention. Analysis of outcome data will be undertaken by the Chief Investigator. The PenCTU will recode participant trial identifiers to ensure analyses are undertaken without knowledge of trial group allocation to reduce any potential bias. Moreover, subjective bias will be minimised by the prospectively published statistical analysis plan and by using standard operating procedures for all trial processes.

## Data collection

Data will be entered into the RedCAP database. Table s7 outlines the timeline of data collection throughout the trial.

### Statistical plan

#### Sample size

The aim of the study is to provide estimated rates of feasibility outcomes (recruitment, retention, fidelity). For a feasibility RCT designed with 80% power and one-sided 5% alpha, a sample size of 58 participants (29 per treatment arm) is recommended.^22^ Eighty-four participants will be recruited (42 per group). This will allow for a loss to follow-up due to mortality (up to 30%).^6^ This number is based upon recruitment uptake, participant retention and intervention fidelity using a red, amber, green (RAG) system as per best practice guidelines (Table s8) and in consultation with the PenCTU and expert opinion (VA, DM).^22^ The RAG system will guide progression to a definitive trial of effectiveness where green (go) and amber (go with changes made) criteria are met. If feasibility criteria are within the red, this means progression to a full trial will only be considered if changes are possible. The RAG criteria have been pre-defined in consultation with expert opinion and will be consulted across the length of the feasibility trial by the key stakeholders (Chief Investigator, PIs, PenCTU, Sponsor, Trial Steering Committee and Trial Management Group).^23^ This estimated sample size aims to ensure all areas of uncertainty that is, feasibility measures, are tested and an appropriate sample population for the qualitative study and mechanistic sub-study is achieved.

#### Analysis of outcomes

A detailed statistical analysis plan has been prospectively published on the ISRCTN registry. Participant flow through the trial will be reported using the CONSORT 2010 statement.^24^ The Chief Investigator will transport the data recorded using RedCAP into SPSS (version 28.0) for analysis. Baseline demographic, clinical characteristics and missing data will be presented to indicate between group differences (Table s9). Medians (interquartile range, range) will be reported for ordinal data, mean (95% confidence intervals) for continuous data and raw count (number, %) for nominal data. For the patient outcome data, descriptive statistics, mean (standard deviation, range) for continuous outcomes where the distribution appears approximately normally distributed (and medians, inter-quartile range, range otherwise) and raw count (%) for categorical outcomes, will be reported. Estimates of effect sizes will be calculated with 95% confidence intervals (CIs). Factors that may confound findings will be descriptively reported in both groups for example, sedation.

## Monitoring

### Protocol adherence

This trial will be subject to monitoring by University Hospitals Plymouth NHS Trust under their remit as sponsor to ensure adherence to the UK Policy Framework for Health and Social Care Research (2017).^25^ The PenCTU will carry out central data monitoring. Deviations recorded in RedCAP will notify the PenCTU. These will be reviewed, and onwards reporting made where appropriate.

### Safety

During the intervention, if the participant deteriorates, the intensity of the intervention will be reduced. If there is no change within 5 min, the intervention will be stopped. The local site ICU medical team will be notified, and the patient reviewed. The intervention will not be restarted without the participant meeting the safety criteria and ICU team approval.

Participants will be monitored throughout the intervention by the Chief Investigator/site physiotherapist/ICU rehabilitation team member and ICU bedside nurse. All safety events will be reported in accordance with the HRA guidelines for non-clinical trials of investigational medicinal products.^26^ Any untoward and unexpected medical occurrence or effect that results in death, is life-threatening, requires hospitalisation or prolongation of existing inpatients’ hospitalisation, results in persistent or significant disability or incapacity, is a congenital anomaly or birth defect, is a significant or important medical event will be defined as a serious adverse event (SAE). Moreover, adverse events (AEs) directly related to the intervention (i.e. that occur during and for 30-min after the intervention) will be recorded and reported (Supplemental Table s10) by the authorised local site staff.

## Ethics

This trial has received ethical approval from the Health Research Authority (HRA) and Oxford South C Research Ethics Committee (REC reference: 24/SC/0096). This research will be conducted in full conformity with relevant regulations and with the UK Policy Framework for Health and Social Care Research (2017), which have their basis in the Declaration of Helsinki.^25^

### Consent

The trial participant/consultee information sheets (PIS) have been co-produced with, and the participant consent/consultee declaration form in consultation with, PPIE representatives to ensure inclusivity and sensitivity of language. Patients requiring IMV are usually sedated and/or extremely unwell, therefore tend to lack capacity at the time when consent will be sought for this trial. Thus, the standard model of consent will be followed as per the HRA guidelines.^27^ This is described below.

### Personal consultee

Agreement to participate will be sought from a personal consultee in the case where the participant is found to not have capacity for example, unable to recall, understand and retain the information provided. The HRA defines a personal consultee as a person who is ‘engaged in caring for the participant (not professionally or for payment) or interested in his/her welfare and, is prepared to be consulted’.^27^ The personal consultee will be expected to inform the research team on the patient’s wishes if they were able to give consent and if the potential participant should take part. The personal consultee is expected to only provide advice to the research team. The responsibility to enrol the participant will be up to the research team. The personal consultee will be given a full detailed explanation (verbal and written) of the research including each component, time to consider the decision and offered the relevant PIS. They will be made aware that the potential participant can withdraw from the research or specific components of the research, at any point, without giving a reason for their decision.

### Nominated consultee

The research team will nominate a professional consultee if there is no appropriate person identified (in-person or by telephone) or willing to act as a personal consultee. A nominated consultee is defined as ‘a person independent of the research’.^27^ The procedure for obtaining agreement will be followed as described above.

### Retrospective consent

If the participant regains capacity during the trial period, the research team will inform them of their enrolment in the trial. The participant will be identified to have capacity if they are able to ‘understand the information about the research, retain the information, use or weigh up the information and communicate their decision’.^27^ The procedure for obtaining consent will be followed as described previously. The participant will be informed that their decision will not impact the quality of their care.

### Trial management committees

The responsibility for the management of the FRECycl-D trial is that of the trial management group (TMG). The TMG comprises of the Chief Investigator, principal investigators, ICU research teams, sponsor representative, a PPIE representative and PenCTU team. Moreover, a trial steering committee (TSC) will provide independent oversight of the trial, including data monitoring in accordance with the DAMOCLES Charter.^28^

## PPIE

The National Institute for Health and Care Research (NIHR) recommendations have guided PPIE across all stages of this trial.^29^ The reporting of PPIE in this research has been informed by the GRIPP2 short form checklist.^30^ The NIHR defines PPIE as ‘research being carried out with or by members of the public rather than to, about or for them’. Moreover, the NIHR defines the term ‘public’ to mean patients, potential patients, carers and members of the public that influence and contributes towards research.^30^ This research was designed in consultation with PPIE representatives with experience of delirium as patients in the ICU or their relatives. One PPIE representative is a member of the research team and has provided expertise into the design of the feasibility RCT and qualitative evaluation. Four public advisory group (PAG) members provided input into the intervention, methods and outcome measures. One public member co-produced the relevant participant/consultee information sheet (PIS) and all PAG members reviewed the PIS and consent/declaration forms. The PAG and additional PPIE members will continue to be included throughout the research process.

## Conclusion

The FRECycl-D trial will generate important information about a method of early mobilisation for patients at high risk of developing delirium in the ICU. Moreover, it will explore an appropriate outcome measure of delirium in a critically ill patient population receiving IMV. This programme of research will provide the framework for a future definitive RCT. The FRECycl-D trial has been successfully adopted on the NIHR CRN portfolio and is currently registered on the NIHR Associate Principal Investigator scheme.

## Supplemental Material

sj-doc-1-inc-10.1177_17511437251400612 – Supplemental material for Does in-bed cycling delivered within 48 hours of mechanical ventilation, reduce the occurrence of delirium in critically ill patients: A mixed-methods feasibility randomised controlled trial protocol Supplemental material, sj-doc-1-inc-10.1177_17511437251400612 for Does in-bed cycling delivered within 48 hours of mechanical ventilation, reduce the occurrence of delirium in critically ill patients: A mixed-methods feasibility randomised controlled trial protocol by Jacqueline Bennion, Mark Hudson, Mary Hickson, Victoria Allgar, Bridie Kent, David McWilliams and Daniel Martin in Journal of the Intensive Care Society
