# Supplementary material for: Does in-bed cycling delivered within 48 hours of mechanical ventilation, reduce the occurrence of delirium in critically ill patients: A mixed-methods feasibility randomised controlled trial protocol
Source: J Intensive Care Soc. 2025 Dec 8;27(1):90–7. doi: 10.1177/17511437251400612 (PMC12685707; doi:10.1177/17511437251400612)
Supplement: sj-doc-1-inc-10.1177_17511437251400612 – Supplemental material for Does in-bed cycling delivered within 48 hours of mechanical ventilation, reduce the occurrence of delirium in critically ill patients: A mixed-methods feasibility randomised controlled trial protocol [file sj-doc-1-inc-10.1177_17511437251400612.doc]

**SUPPLEMENTARY FILE**

**Trial title**: Does in-bed **cycl**ing delivered within 48 hours of mechanical ventilation, r**e**duce the occurrence of **d**elirium in critically ill patients**:** A mixed-methods **f**easibility **r**andomised controlled trial protocol.

Contents

[Table. s1 Listed amendments 2](#__RefHeading___Toc207268073)

[Figure s1. In-bed cycling protocol 6](#__RefHeading___Toc207268074)

[International recommendations of safety criteria for in-bed and out of bed mobilisation of critically ill patients. 8](#__RefHeading___Toc207268075)

[Table s2. Description of the traffic light system approach to the safety criteria 8](#__RefHeading___Toc207268076)

[Table s3. Traffic light system of safety considerations for the respiratory system. 8](#__RefHeading___Toc207268077)

[Table s4. Traffic light system of safety considerations for the cardiovascular system. 9](#__RefHeading___Toc207268078)

[Table s5. Traffic light system of safety considerations for the neurological system. 11](#__RefHeading___Toc207268079)

[Table s6. Traffic light system of additional safety considerations for other conditions and symptom presentation in the ICU. 12](#__RefHeading___Toc207268080)

[Table s7. Timeline of data collection 13](#__RefHeading___Toc207268081)

[Table s8. RAG system to guide progression to a definitive trial 14](#__RefHeading___Toc207268082)

[Table s9. Baseline Characteristics 15](#__RefHeading___Toc207268083)

[Table s10. Expected Event (related to the intervention) 17](#__RefHeading___Toc207268084)

# Table. s1 Listed amendments

| **Amendment No.** | **Protocol Version No.** | **Date issued** | **Author(s) of changes** | **Details of changes made** |
| --- | --- | --- | --- | --- |
| **SA1** | **3.0** | **22.05.2024** | **CI** | **Blinding of outcome assessments: The PenCTU outlined that it will now not be possible to ensure blinding of outcome assessments due to the CI carrying out the intervention and all outcome measures in accordance with their PhD training. Therefore, the FRECycl-D trial will not be blinded.** |
|  |  |  |  | **Randomisation: Unfortunately, the description of randomisation in the FRECycl-D trial was overlooked. Randomisation will be stratified according to site.** |
|  |  |  |  | **Venous Blood Gas (VBG) and Arterial Blood Gas (ABG): The FRECycl-D PIS and IRAS specified that 6mls of blood will be collected per consenting participant per VBG and per ABG. Apologies, this did not include the recovery timepoint i.e., 1ml ABG and 1ml VBG. This means 7mls of blood per participant per VBG and per ABG will be collected.** |
|  |  |  |  | **Statistical analysis software: The University of Plymouth is not licensed for students to access the STATA software for statistical analysis therefore SPSS will be used instead. This should not impact upon the quality of the statistical analysis of the FRECycl-D trial.** SPSS is an appropriate alternative for feasibility trials. Its use will not compromise statistical integrity. |
|  |  |  |  | **The use of the APACHE II score to measure severity of illness at admission requires measurement of the participants temperature from their rectum. This is not routine clinical practice in the ICU. Therefore, the SOFA score will be used instead. This tool is a validated outcome measure of the severity of illness and uses data routinely collected in clinical practice. The SOFA score is being increasingly recognised as a more optimal tool than the more well known research tool, the APACHE II score.** |
|  |  |  |  | **Due to the length of the PIS, a lay summary leaflet will be used initially alongside the PIS for potential participants. This has been discussed in the REC meeting and with the FRECycl-D trial PPI representatives. The suggestion of the lay summary leaflet was positively received by all parties.** |
| **NSA 01** | **4.0** | **26.07.2024** | **CI** | **The supplier of the Near Infrared Spectroscopy (NIRS) device (INVOSTM 5100C) has offered an updated model. The INVOS TM 7100 is more user friendly and provides the options of downloading data in real-time without the need for mapping or costly licenses. It also comes with smaller sensor pads for the patient's forehead. Therefore these are not restricted to the patients forehead size, shape and hairline. The updated device and training comes at no extra cost or resource use. Moreover the device has been validated for clinical practice.** |
|  |  |  |  | **The University of Birmingham Immunology Service has found during another study that the Cytometric Bead Array technique has demonstrated extremely inconsistent results. Therefore the diagnostic laboratory has recommended using a fluidic-based assay technique on the Luminex platform to ensure the analysis of the biomarkers is more reliable.** |
| **NSA 02** | **5.0** | **20.08.2024** | **CI** | **Following recommendations by the Trial Steering Committee table 5.0 section 7.3 has been revised to ensure the randomisation timepoint and baseline data are distinguishable e.g., that baseline data will be collected (retrospectively) prior to randomisation i.e., at ICU admission. This is to ensure consistency of data collection across both sites.** |
|  |  |  |  | **Following discussion with the PenCTU and Trial Management Group it was agreed that the process in which participants resume in the trial according to their allocated group if they are re-admitted to the ICU or deteriorate requiring invasive mechanical ventilation again within 90 days of randomisation should be described in further detail. This is to aid consistent data collection across sites and the further development of the trial database to capture these data.** |
|  |  |  |  | **The recently published ICNARC Case Mix Programme data for the period of 2022-2023, suggests that the median length of stay in the ICU is 3.0 days. This appears to be comparable to the local ICU site turnover. Currently the additional venous blood samples are collected at days 0,7,14 from randomisation. This has now been revised in relation to these recently published data. Therefore, the additional venous blood samples will be collected at days 0,3,5 from randomisation. Where the timepoints fall on a weekend, the samples will be collected at the nearest weekday. This is to maximise the likelihood of collecting these data prior to the patient’s discharge and accommodate the lack of weekend cover.** |
|  |  |  |  | **The safety criteria as described in the appendices have been reviewed by the local site Medical Director and the Director of Studies to ensure the criteria are relevant to the population concerned. Details have been more clearly specified and additional information added (k+, double-lumen Endotracheal Tube and airway blocks). This is as advised by the authors of the safety criteria (see references) where they recommend the use of the safety criteria as a guide only and treatment should be made specific to the patient population concerned.** |
| **NSA 03** | **6.0** | **19.12.2024** | **CI** | **Dr Jessie Welbourne is the current PI for the Derriford site. Unfortunately, due to her expanded clinical-academic role and external commitments, she is no longer able to fulfil her role as PI. Therefore, Professor Dan Martin will take her place.** |
| **NSA 04** | **7.0** | **29.04.2025** | **CI** | **The addition of a 3^rd^ site, Blackpool Teaching Hospital NHS Foundation Trust.** |
| **NSA 05** | **8.0** | **11.06.2025** | **CI** | **Feedback from the SWP Research Community meeting queried whether the inclusion criteria of 'expected to remain >72 hours mechanically ventilated' should be revised. Site screening logs were reviewed. A total of 74 patients were not eligible due to this criterion. The Intensive Care National Audit and Research Centre (ICNARC) Case Mix Programme recent quarterly reports for each site were also reviewed. This showed a median of <3 days IMV at Torbay and Derriford Hospital and a median of 3.2 days at Blackpool hospital. Therefore, the inclusion criteria have been changed to 'expected to remain >24 hours mechanically ventilated'. This change will optimise feasibility objectives (mainly recruitment and subsequently the remaining feasibility objectives) of participants at all sites.** |

# Figure s1. In-bed cycling protocol


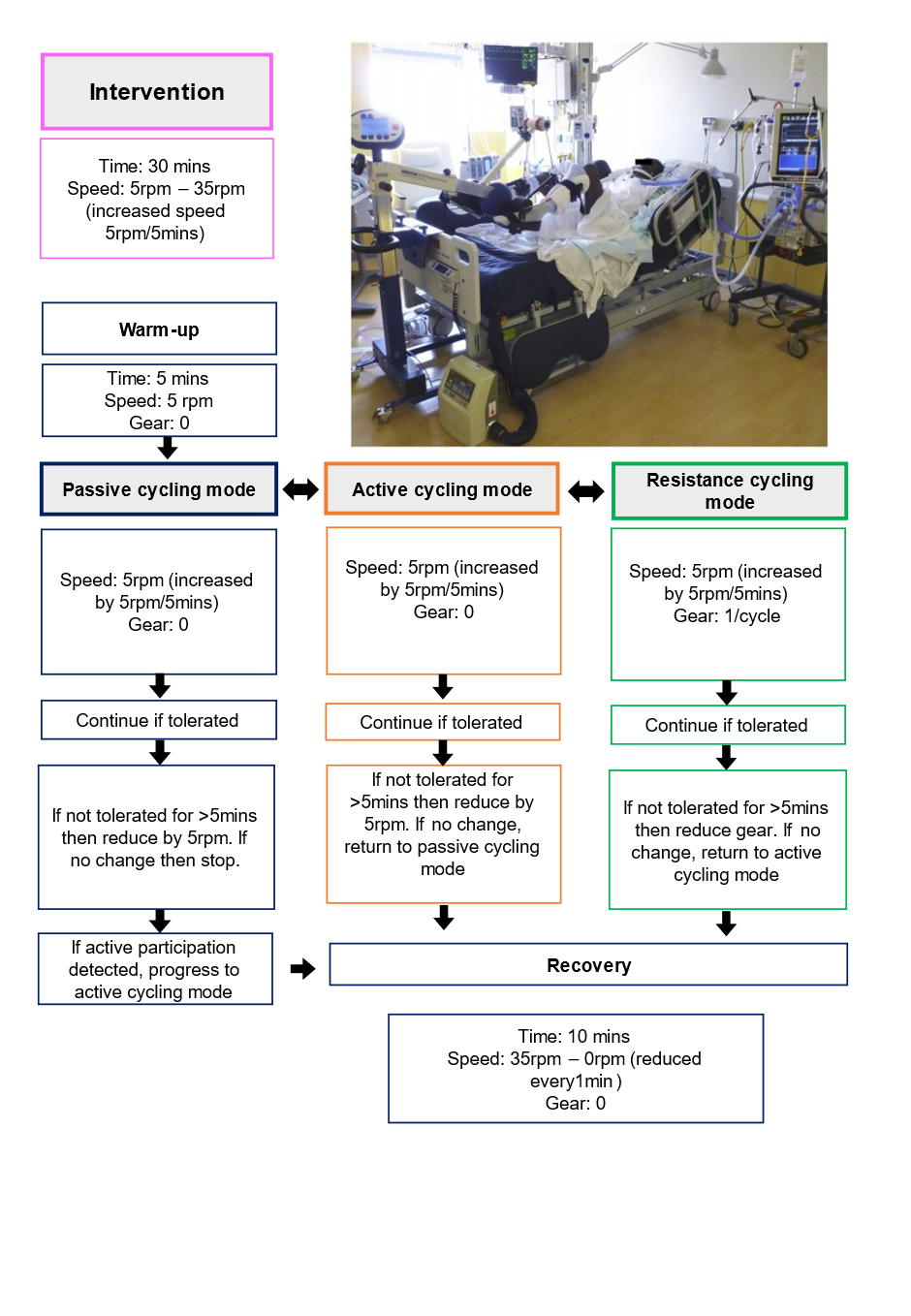


- The patient will be positioned in the in-bed cycling device by the physiotherapist/ICU rehabilitation team member and the bedside nurse.
- The 45-minute intervention includes a warm-up (5 minutes) and recovery (10 minutes).
- The protocol involves progressive intensity starting at 5 revolutions per minute (rpm) to 35 rpm over 30 minutes.
- The cycle device will be programmed to use a progressive protocol between passive, active and resistance modes.
- The device allows participants to take over from the device to activate the different modes. This means the participant will be able to activate and progress between passive, active and resistance modes.
- Conversely, the device allows participants to regress e.g., if fatigue develops. This means the device will take over from the participant to continue in the passive mode.
- The site physiotherapist/ICU rehabilitation team member will document the recorded data from the device for each session.

# International recommendations of safety criteria for in-bed and out of bed mobilisation of critically ill patients.

## Table s2. Description of the traffic light system approach to the safety criteria


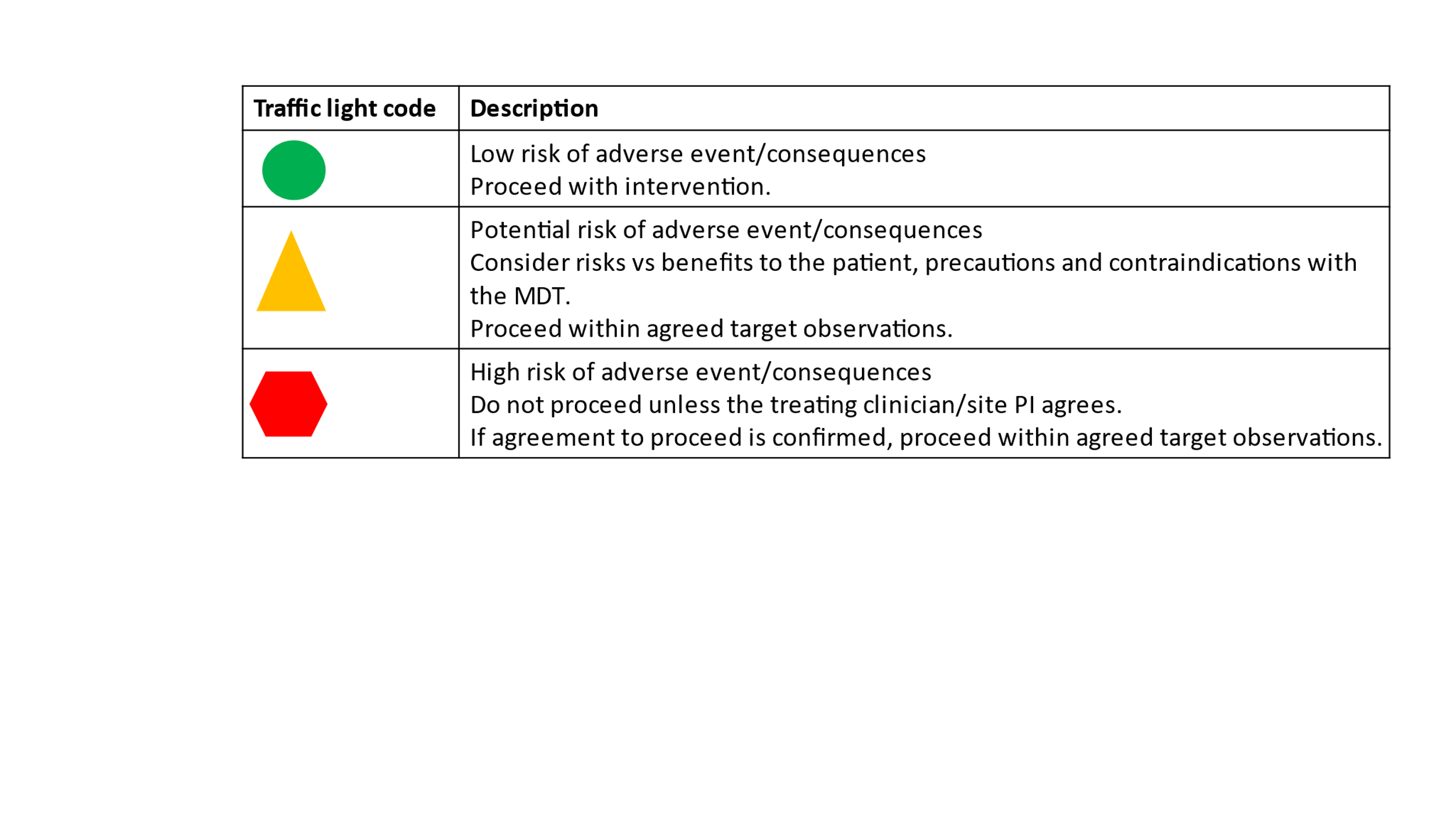


## Table s3. Traffic light system of safety considerations for the respiratory system.

| **RESPIRATORY CONSIDERATIONS** | **TRAFFIC LIGHT CODE** |
| --- | --- |
| **Intubation** | |
| Endotracheal tube | 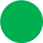 |
| Tracheostomy/laryngectomy tube | 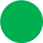 |
| Double-lumen Endotracheal tube | 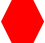 |
| Airway nerve block | 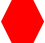 |
| **Respiratory Parameters** | |
| Fraction of inspired Oxygen (FiO2) | |
| ≤ 0.60 | 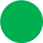 |
| > 0.60 | 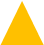 |
| Percutaneous Oxygen Saturation (SPO2) | |
| ≥ 90% | 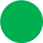 |
| < 90% | 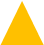 |
| **Respiratory Rate (bpm)** | |
| ≤ 30 bpm | 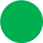 |
| > 30 bpm | 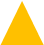 |
| **Ventilation** | |
| PEEP (Positive End Expiratory Pressure) | |
| ≤ 10 cmH2O | 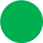 |
| > 10 cmH2O | 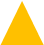 |
| Ventilator desynchrony | 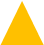 |
| **Rescue therapies** | |
| Nitric Oxide | 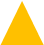 |
| Prostacyclin | 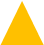 |
| Prone positioning | 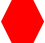 |

## Table s4. Traffic light system of safety considerations for the cardiovascular system.

| **A.** | |
| --- | --- |
| **CARDIOVASCULAR CONSIDERATIONS** | **TRAFFIC LIGHT CODE** |
| **Blood Pressure** | |
| Intravenous antihypertensive therapy for hypertensive emergency. | 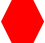 |
| MAP (Mean Arterial Pressure) | |
| Below target range and causing symptoms. | 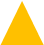 |
| Below target range despite support (vasoactive and/or mechanical). | 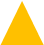 |
| Greater than lower limit of target range while receiving no support or *low-level* support. | 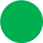 |
| Greater than lower limit of target range while receiving *moderate level* support. | 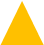 |
| Greater than lower limit of target range while receiving *high level* support. | 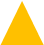 |
| Known or suspected severe pulmonary hypertension | 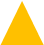 |
| **Cardiac Arrythmias** | |
| **Bradycardia** | |
| Requiring pharmacological treatment e.g., isoprenaline or awaiting *emergency* pacemaker insertion. | 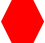 |
| Not requiring pharmacological treatment and not awaiting *emergency* pacemaker insertion. | 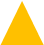 |
| Transvenous or epicardial pacemaker | |
| Dependent rhythm | 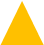 |
| Stable underlying rhythm | 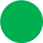 |
| **B.** | |
| **CARDIOVASCULAR CONSIDERATIONS** | **TRAFFIC LIGHT CODE** |
| **Cardiac Arrythmias** | |
| Any stable tachyarrhythmia | |
| Ventricular rate >150 bpm | 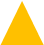 |
| Ventricular rate 120-150 bpm | 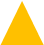 |
| Any tachyarrhythmia < 120 bpm | 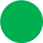 |
| **Devices** | |
| Femoral intra-aortic balloon pump (IABP) | 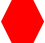 |
| ECMO: | |
| Femoral or subclavian (not single bicaval dual lumen cannulae)  **Cycling may be contraindicated in the leg of the cannulae insertion. Their position may need to be modified.* | 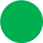 |
| Single bicaval dual lumen cannulae inserted into a central vein | 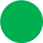 |
| Ventricular assist device | 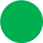 |
| Pulmonary artery catheter or other continuous cardiac output monitoring device | 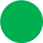 |
| **Other cardiovascular considerations** | |
| Shock of any cause with lactate > 4mmol/L | 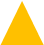 |
| Acute potassium (K+) ≥ 6.0 mmol/L | 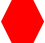 |
| Known or suspected acute DVT/PE | 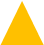 |
| Known or suspected severe aortic stenosis | 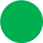 |
| Cardiac ischaemia (defined as ongoing chest pain and/or dynamic *ECG* changes | 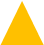 |

## Table s5. Traffic light system of safety considerations for the neurological system.

| **NEUROLOGICAL CONSIDERATIONS** | **TRAFFIC LIGHT CODE** |
| --- | --- |
| **Level of consciousness (RASS)** | |
| RASS -1 to +1: Patient drowsy, calm, or restless | 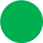 |
| RASS -2 to +2: Patient lightly sedation or agitated | 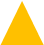 |
| RASS < -2: Patient unrousable or deeply sedated | 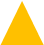 |
| RASS > +2: Patient very agitated or combative | 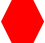 |
| **Delirium** | |
| Delirium tool e.g., Confusion Assessment Method for the ICU (CAM-ICU) negative | 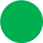 |
| Delirium tool positive and able to follow simple commands | 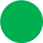 |
| Delirium tool positive and not able to follow commands | 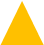 |
| **Intracranial pressure (ICP)** | |
| Active management of intracranial hypertension with ICP not in desired range. | 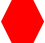 |
| Intracranial monitoring without active management of intracranial hypertension. | 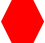 |
| **Other neurological considerations** | |
| Craniectomy | 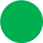 |
| Open lumbar drain (not clamped) | 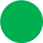 |
| Subgaleal drain | 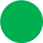 |
| Spinal precautions (pre-clearance or fixation) | 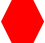 |
| Acute spinal cord injury | 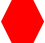 |
| Subarachnoid haemorrhage with unclipped aneurysm | 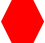 |
| Vasospasm post-aneurysmal clipping | 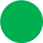 |
| Uncontrolled seizures | 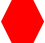 |

## Table s6. Traffic light system of additional safety considerations for other conditions and symptom presentation in the ICU.

| **OTHER CONSIDERATIONS** | **TRAFFIC LIGHT CODE** |
| --- | --- |
| **Surgical** | |
| Unstable/unstabilised major fracture (pelvic, spinal, lower limb) | 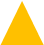 |
| Large open surgical wound (chest/sternum, abdomen) | 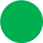 |
| **Medical** | |
| Known uncontrolled active bleeding | 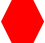 |
| Suspicion of active bleeding or increased bleeding risk | 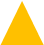 |
| Patient is febrile with a temperature exceeding acceptable maximum despite active physical or pharmacological cooling management | 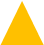 |
| Active hypothermia management | 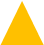 |
| **Other considerations** | |
| ICU-acquired weakness | 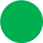 |
| Continuous renal replacement therapy (including femoral dialysis catheters). | 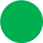 |
| Venous or arterial femoral catheters | 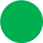 |
| Femoral sheaths | 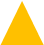 |
| All other drains and attachments e.g.,  Nasogastric tube  Central venous catheter  Pleural drain  Wound drain  Intercostal catheter  Urinary catheter | 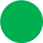 |

# Table s7. Timeline of data collection

|  |  | **Timepoint** | | | | | |
| --- | --- | --- | --- | --- | --- | --- | --- |
|  | **Screening** | | **Baseline**  **(ICU admission)** | **Randomisation** | **Day 0-14** | **Day 30** | **Day 90** |
| Eligibility Screen | x | |  |  |  |  |  |
| Informed consent/agreement | x | |  |  |  |  |  |
| Patient contact details |  | |  |  |  |  |  |
| Randomisation |  | |  | x |  |  |  |
| Demographics |  | | x |  |  |  |  |
| Medical history |  | | x |  |  |  |  |
| Feasibility data |  | |  | x | x | x | x |
| Secondary outcomes |  | |  | x | x | x | x |
| AE/SAE data |  | |  |  | x | x | x |
| Interview |  | |  |  | x | x | x |
| **Follow-up** |  | | | | | | |
| QoL, (EQ-5D-5L, SF-36, proxy-EQ-5D-5L) |  | |  |  |  |  | x |
| Pain, (SF-36) |  | |  |  |  |  | x |
| Physical function, (6MWT). |  | |  |  |  |  | x |
| Cognition, (MOCA) |  | |  |  |  |  | x |
| Delirium, (FAM-CAM) |  | |  |  |  |  | x |
| Time to delirium resolution |  | |  |  |  |  | x |

# Table s8. RAG system to guide progression to a definitive trial

|  | Green  (Go – proceed with RCT) | Amber  (Amend – proceed with changes) | Red  (Stop – do not proceed unless changes are possible) |
| --- | --- | --- | --- |
| Proposed recruitment rate (%) | >20% | 10-20% | <10% |
| Retention rate (%) | ≥85% | 65-85% | <65% |
| Intervention fidelity (%) | ≥75% | 50-75% | <50% |

# Table s9. Baseline Characteristics

| **Age** |
| --- |
| **Sex at birth:**  Male  Female  Unknown |
| **Ethnicity:**  Asian or Asian British   - Indian - Pakistani - Bangladeshi - Chinese - Any other Asian background   Black, Black British, Caribbean or African   - Caribbean - African - Any other Black, Black British, or Caribbean background   Mixed or multiple ethnic groups   - White and Black Caribbean - White and Black African - White and Asian - Any other Mixed or multiple ethnic background   White   - English, Welsh, Scottish, Northern Irish or British - Irish - Gypsy or Irish Traveler - Roma - Any other White background   Other ethnic group   - Arab - Any other ethnic group |
| **Comorbidities**  **Charleston Comorbidity Index)** |
| **Dependency Prior to ICU admission** **(Clinical Frailty Scale):**   1. Very fit 2. Fit 3. Managing well 4. Living with very mild frailty 5. Living with mild frailty 6. Living with moderate frailty 7. Living with severe frailty 8. Living with very severe frailty 9. Terminally ill |
| **Body Mass Index (BMI) (kg/m^2^)** |
| **Reason for ICU admission:**  Pneumonia  Respiratory failure  Surgical  Trauma  Traumatic brain injury  Liver failure  Renal failure  Neurological disorder |
| **Severity of illness (SOFA score)** |

# Table s10. Expected Event (related to the intervention)

| **Expected event** | **Description** |
| --- | --- |
| Acute arterial desaturation | A peripheral oxygen saturation (SpO_2_) below consistently below 80% |
| Hypotension (despite appropriate treatment) | A mean arterial pressure (MAP) below 50 mmHg |
| Bradycardia | HR less than 40 bpm or 50% below baseline |
| Atrial Fibrillation (AF) | New onset AF (if previously in sinus rhythm) or a 50% increase in heart rate (HR) from baseline in established AF |
| Sinus Ventricular Tachycardia (SVT) | New onset of SVT |
| Sinus Tachycardia | Increase in HR to >150 bpm or an increase of > 50% from baseline HR |
| Raised intracranial Pressure (ICP) | ICP exceeds target range |
| Bruising on lower limbs at site of the in-bed cycling device |  |
